# Competition for gradient-free tuning of large language models: approaches, results, current challenges and future directions

**DOI:** 10.1093/nsr/nwad124

**Published:** 2023-05-04

**Authors:** Tingfeng Cao, Liang Chen, Dixiang Zhang, Tianxiang Sun, Zhengfu He, Xipeng Qiu, Xing Xu, Hai Zhang

**Affiliations:** School of Software Engineering, South China University of Technology, China; School of Software Engineering, South China University of Technology, China; School of Software Engineering, South China University of Technology, China; School of Computer Science, Fudan University, China; School of Computer Science, Fudan University, China; School of Computer Science, Fudan University, China; School of Computer Science and Engineering, University of Electronic Science and Technology of China, China; Pazhou Laboratory (Huangpu), China; School of Mathematics, Northwest University, China; Pazhou Laboratory (Huangpu), China

## Abstract

This perspective presents a brief overview of the background of Gradient-free tuning for large language models competition, the championship scheme, as well as the challenges and future directions.

## PROBLEM

Recent years have witnessed the rapid progress of self-supervised language models (LMs) [1], especially large language models (LLMs) [2]. LLMs not only achieved state-of-the-art performance on many natural language processing tasks, but also captured widespread attention from the public due to their great potential in a variety of real-world applications (e.g. chatbots, search engines, writing assistants, etc.) through providing general-purpose intelligent services. A few of the LLMs are becoming foundation models, an analogy to infrastructure, that empower hundreds of downstream applications. Currently, most competitive LLMs such as OpenAI’s GPT-3 [2] are released as services, allowing users to access these powerful models through black-box APIs. In such a scenario, termed *language-model-as-a-service* (LMaaS) [3], how to solve downstream tasks through black-box APIs is a challenging problem.

The challenge mainly lies in the invisibility of the model weights and their gradients, making conventional backpropagation-based training techniques infeasible. As an alternative, derivative-free optimization (DFO) does not depend on gradients but relies only on function values, i.e. the results returned by the black-box API. However, LLMs have tens or even hundreds of billions of parameters and DFO suffers from a slow convergence rate when the dimensionality of the search space is high. It has been demonstrated that combining parameter-efficient tuning and DFO methods can effectively drive LLMs through their black-box APIs to solve a variety of classification tasks under few-shot settings [3,4]. Despite their success, these methods, named *black-box tuning*, still lag behind backpropagation on some difficult tasks such as many-label classification tasks and entailment tasks in terms of accuracy and efficiency.

The *1st Competition for Gradient-Free Tuning of Large Language Models*, organized within the Guangdong-Hong Kong-Macao Greater Bay Area International Algorithm Case Competition, is one of the first attempts to encourage the development of this promising line of research. The main objectives of the competition were as follows.

- Invite the community to develop derivative-free optimization algorithms for large language models.
- Invite the community to work on solutions that can effectively and efficiently use large language models deployed as services.
- Provide the first opportunity for a standard and comprehensive evaluation on a common hardware platform and a shared set of tasks and metrics for a fair comparison.

The competition includes five public natural language understanding tasks: topic classification (DBPedia-14), sentiment classification (SST-2), textual entailment (SNLI), question matching (QQP) and question-answering matching (QNLI); see the online supplementary material for further details. The multi-label tasks (i.e. SNLI and DBPedia-14) are evaluated with the macro-F1 metric. The rest are binary classification tasks and are evaluated with accuracy.

## ALGORITHM

### Background

**Prompt-based learning.** Prompt-based learning is a new paradigm that converts downstream tasks into (masked) language modeling, reducing the gap between pre-training and downstream tasks [2,5]. For example, for a sentiment analysis sample, ‘*A fantastic movie*’, we can modify the input to ‘*A fantastic movie. It was*[MASK].’, and let the language model predict the masked word as ‘*great*’ or ‘*terrible*’.

**Black-box tuning.** Black-box tuning (BBT) [3] is a gradient-free framework that optimizes continuous prompt prepended to the input text merely by means of black-box inference APIs. In particular, it optimizes a low-dimensional vector using the covariance matrix adaptation evolution strategy (CMA ES) [6] and then projects it to a higher-dimensional space to obtain the final continuous prompt. BBT adopts a prompt-based learning paradigm that reuses the masked language modeling head of the pre-trained language model.

**BBTv2.** BBTv2 is an improved version of black-box tuning. Instead of prepending the continuous prompt merely to the input text, BBTv2 prepends to hidden states of every layer and proposes a divide-and-conquer algorithm [4] to alternately optimize the injected prompts from the bottom to the top. In addition, it uses normal distributions with model-related standard deviations to generate random projections, making the distribution of continuous prompts closer to that of the word embedding or hidden states of the model.

### The proposed solution

**Figure 1.**
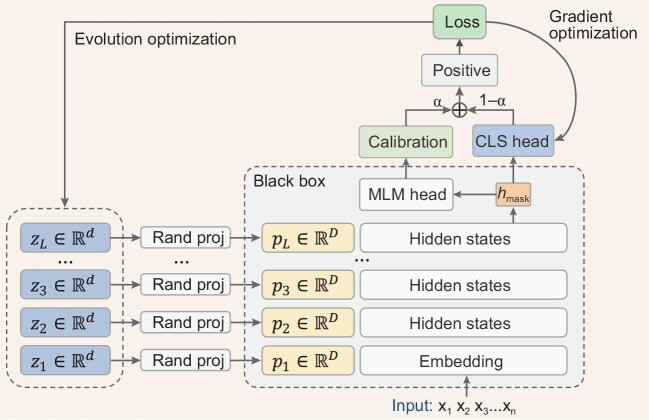
Overview of the winning solution. Here *h*_mask_ denotes the hidden states of the masked token, *d* and *D* refer to the dimensionality of the low-dimensional continuous prompt *z* and the hidden size of the language model, respectively.

In this section, we present the solution proposed by the champion team, which is based on BBTv2 with the following several improvements. The overall illustration of the proposed solution is shown in Fig. 1.

**Calibration.** The predictions of pre-trained LMs are usually biased due to the word frequency of the pre-training corpus [7]. For example, given a template with an empty input ‘*It was*[MASK]’, LMs tend to predict with a higher probability ‘*great*’ rather than ‘*terrible*’ on the masked position. Therefore, we use a calibration module to calibrate the predicted probabilities over words so that the output probabilities of the words representing each label are approximately equal.

In particular, all training samples are formatted with prompt and then fed into the pre-trained LM. We average the output logits of the masked position across all the samples. The obtained result can be formulated into a diagonal matrix and inverted as


(1) \begin{eqnarray*} {\bf W} = \lambda \cdot \text{diag}\bigg (\frac{1}{N} \sum \limits _{i=1}^{N} {\bf q}^i\bigg )^{-1}, \end{eqnarray*}

where *N* is the total number of samples, *q*^*i*^ is the logits corresponding to sample *i* and λ is the scaling factor that scales the logits back to their original scale, i.e.


(2) \begin{eqnarray*} \lambda = \frac{1}{N \cdot K} \sum _{i=1}^{N} \sum _{j=1}^{K} {\bf q}_j^i, \end{eqnarray*}

where *K* is the number of classes in the classification task. In this way, the calibrated probability distribution can be obtained by **p**_1_ = softmax(**Wq**).

**Integrating with the feature-based method.** In the scenario of LMaaS, a straightforward way to use the black-box model is to extract the features of the samples and train a local classifier with gradient descent. In this way, the pre-trained LM serves as a feature extractor and therefore the expensive backpropagation through the large LM can be avoided. However, despite its lower cost (one sample for one inference API), its performance is much lower than BBT [3] and BBTv2 [4]. In this solution, we combine the feature-based method and black-box tuning. On the one hand, we use BBTv2 to fine-tune a small portion of parameters of the model. On the other hand, we use the final hidden states of the [MASK] token as features to train a classifier:


\begin{eqnarray*} {\bf{ p}}_2 = {\text{softmax}} (f({\bf{h}}_{\text{mask}})). \end{eqnarray*}

Here *f* is the local classifier optimized directly by gradient descent and *h*_mask_ is the hidden states of the [MASK] token.

Furthermore, it was observed in the experiments that the two approaches are complementary and therefore we combine the two approaches and optimize them jointly as


\begin{eqnarray*} {\bf p} = \alpha \cdot {\bf p}_1 + (1-\alpha ) \cdot {\bf p}_2, \end{eqnarray*}

where α is a hyper-parameter to balance the two approaches. In practice, we set α = 0.5. By integrating the feature-based method, we achieved not only faster convergence but also improved accuracy. We also found in our experiments that the performance improvement of our method does not mainly come from the weighted output of the two approaches, but rather the two methods share the hidden states of the [MASK] token **h**_mask_, allowing **h**_mask_ to learn a better semantic representation.

**Training protocol.** In the proposed solution, there are two groups of parameters to be optimized: (1) the low-dimensional continuous prompt **z** to be optimized by the CMA ES and (2) the local classifier *f*_**θ**_ to be optimized by gradient descent. To handle the two optimization problems, we propose an alternating joint optimization (AJO) algorithm. As detailed in Algorithm 1 below, the local classifier *f* is first optimized using gradient descent, followed by CMA ES combined with the divide-and-conquer algorithm [4] to optimize the parameters **z**_*l*_ corresponding to the low-dimensional prompt at layer *l*. We alternate the above process until convergence.

**Algorithm 1.** AJO algorithm

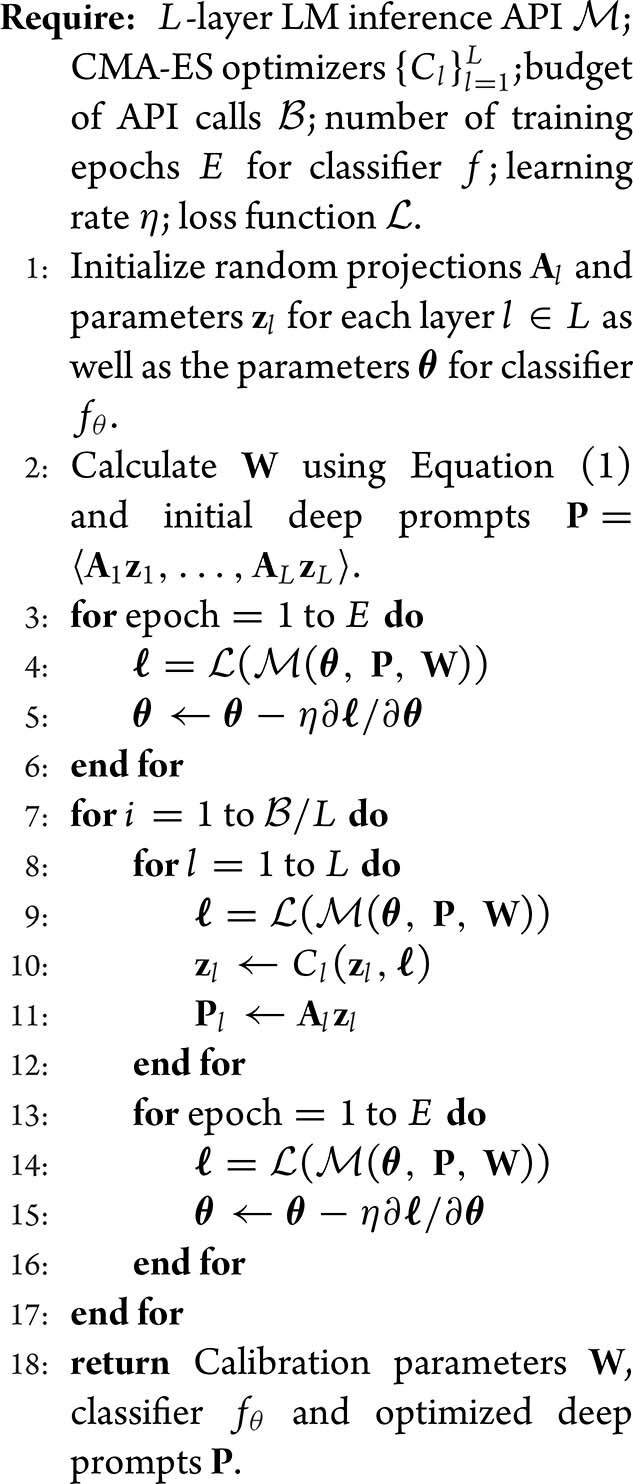

## EVALUATION

In this section, we show the results of the competition for this track in detail. Additionally, we discuss the champion’s method from innovation and application aspects.

### Implementation details

The competition uses RoBERTa-Large as the base model and our learning rate η is set to 3 × 10^−4^; the training epoch *E* is set to 3. For SST-2 and DBPedia, the budget of API calls $\mathcal {B}$ is 3000; for QQP, QNLI and SNLI, the budget of API calls $\mathcal {B}$ is 8000.

### Results

The results of the competition are reported in the online supplementary material; we consider BBTv2 [4] as a strong baseline. By comparison, the champion’s method consistently improves the results on all the datasets and achieves an average absolution gain of 6.6% in terms of accuracy. We can observe relatively large gains on the topic classification task (DBPedia, which has 14 classes) and hard entailment tasks (QQP, QNLI). However, for some of the more difficult tasks, such as SNLI, it can be seen that the improvement is not significant. In addition, we use the number of API calls to calculate the average speedup ratio. As a result, we find that the proposed method achieves a speedup ratio of about 1.3, which implies a lower training budget and a shorter training time.

### Discussion

In this section we present the program committee’s discussion on the winning approach and the competition results.

- **Innovation.** The proposed solution combines two promising approaches for LMaaS, namely, black-box tuning and feature-based methods, corresponding to zeroth-order and first-order optimization, respectively. Such a combination is demonstrated to be beneficial to both accuracy and efficiency. Furthermore, the winning solution incorporates a calibration module to mitigate the prediction bias, which further improves overall accuracy and the robustness of the prompt design.
- **Application.** The winning approach significantly improves accuracy and reduces training costs when adapting large LMs to downstream tasks through black-box APIs. Such improvements naturally extend the scope of the applications of LMaaS.

## FUTURE DIRECTIONS

The 1st Competition for Gradient-Free Tuning of Large Language Models held at Guangdong-Hong Kong-Macao Greater Bay Area International Algorithm Case Competition aims at promoting the development of gradient-free tuning for large LMs to expand the applications of LMaaS. The champion’s method improves the accuracy on all the datasets over the baseline model by combining calibration and integrating with the feature-based method, achieving a new state of the art. This method allows large LMs deployed on cloud servers to be efficiently adapted to a wide range of downstream tasks with only a small number of training samples. The method requires no access to the model weights and gradients and therefore enjoys great advantages on computation budget and security.

We summarize the current challenges and possible future directions as follows.

- **Accuracy and cost.** The current approach has achieved comparable or even better accuracy than conventional backpropagation-based optimization methods. However, the performance of the current approach on more difficult tasks such as machine reading comprehension and information extraction is still under-explored. In addition to the accuracy, the training cost is another important metric to be considered. A single-minded focus on accuracy is undesirable and a composite metric should be designed to encourage the balance between accuracy and training cost.
- **Generation.** Most of the current approaches focused on classification tasks, while generation tasks such as text summarization, machine translation and dialogue are still untouched. Considering the wide applications of the generation tasks, including some generation tasks in the next competition should be encouraged.
- **Compatibility.** The current competition is limited to prompt-based tuning. All the tunable parameters are in the continuous prompt. We advocate the adoption of new gradient-free tuning frameworks that are compatible with more parameter-efficient tuning approaches such as Adapter [8] and BitFit [9].
- **Security.** The large-scale use of model inference APIs poses several security issues, such as stealing model weights via API calls [10], and privacy issues of user data. The measurement and the approaches to mitigate such issues are worth exploring in the following competitions.

## Supplementary Material

nwad124_Supplemental_File
