# Supplementary material for: Competition for gradient-free tuning of large language models: approaches, results, current challenges and future directions
Source: Natl Sci Rev. 2023 May 4;10(6):nwad124. doi: 10.1093/nsr/nwad124 (PMC10278975; doi:10.1093/nsr/nwad124)
Supplement: nwad124_Supplemental_File [file nwad124_supplemental_file.pdf]

SUPPLEMENT

Datasets

- Topic Classification: DBPedia14 [1]
- Sentiment Classification: SST2 [2]
- Textual Entailment: SNLI [3]
- Question Matching: QQP [4]
- Question-answering Matching: QNLI [5]

Results

Table 1. The results of the competition champion team and the baseline (BBTv2).

Tasks	BBTv2	Champion
SST-2	90.2	91.2
DBPedia	90.0	97.1
QQP	57.4	68.5
QNLI	57.2	68.2
SNLI	55.5	57.9
Avg.	70.0	76.6
Speedup	1.0×	1.3×

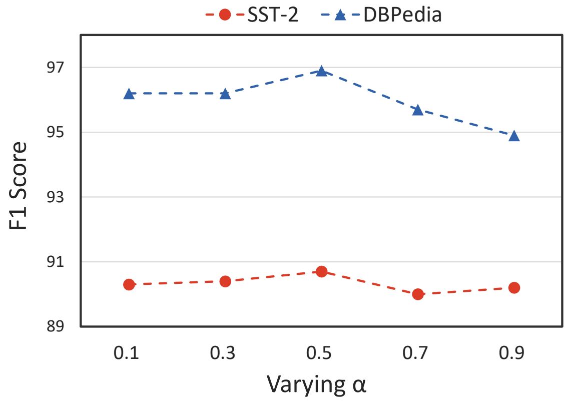

Figure 1. For the parameter analysis of α on SST2 and DBPedia, the publicly available test set is used, which is not consistent with the test set in the competition.

REFERENCES

1. Zhang X, Zhao JJ and LeCun Y. Character-level convolutional networks for text classification. Cortes C, Lawrence ND, Lee DD *et al.*, editors, *Advances in Neural Information Processing Systems 28: Annual Conference on Neural Information Processing Systems 2015, December 7-12, 2015, Montreal, Quebec, Canada* (2015) 649–657.
2. Socher R, Perelygin A, Wu J *et al.* Recursive deep models for semantic compositionality over a sentiment treebank. *Proceedings of the 2013 Conference on Empirical Methods in Natural Language Processing, EMNLP 2013, 18-21 October 2013, Grand Hyatt Seattle, Seattle, Washington, USA, A meeting of SIGDAT, a Special Interest Group of the ACL*, (ACL2013) 1631–1642.
3. Bowman SR, Angeli G, Potts C *et al.* A large annotated corpus for learning natural language inference. Màrquez L, Callison-Burch C, Su J *et al.*, editors, *Proceedings of the 2015 Conference on Empirical Methods in Natural Language Processing, EMNLP 2015, Lisbon, Portugal, September 17-21, 2015*, (The Association for Computational Linguistics2015) 632–642.
4. Wang Z, Hamza W and Florian R. Bilateral multi-perspective matching for natural language sentences. Sierra C, editor, *Proceedings of the Twenty-Sixth International Joint Conference on Artificial Intelligence, IJCAI 2017, Melbourne, Australia, August 19-25, 2017*, (ijcai.org2017) 4144–4150.
5. Wang A, Singh A, Michael J *et al.* GLUE: A multi-task benchmark and analysis platform for natural language understanding. *7th International Conference on Learning Representations, ICLR 2019, New Orleans, LA, USA, May 6-9, 2019*, (OpenReview.net2019) .
